# AWARENESS AND ATTITUDES ABOUT MEDICAL AID IN DYING IN THE U.S

**DOI:** 10.1093/geroni/igae098.4264

**Published:** 2024-12-31

**Authors:** Elissa Kozlov, Samuel Nemeth, Elizabeth Luth

**Affiliations:** Rutgers University, Piscataway, New Jersey, United States; Rutgers School of Public Health, Piscataway, New Jersey, United States; Rutgers University, New Brunswick, New Jersey, United States

## Abstract

Medical Aid in Dying (MAID) allows terminally ill patients to seek a prescription for medication to end their lives. Despite being legal in 10 states and Washington DC, public awareness of and attitudes towards MAID remain unclear. This study aims to assess the level of awareness of MAID laws and attitudes about MAID in the United States. Using mTurk and Prime Panels, we collected a purposive national sample of n= 2,343 people living in states where MAID is legal and n=1151 living in states where MAID is not legal. The sample was normally distributed for income, education, political affiliation, and had diverse religious representation. We over sampled for adults living in MAID states and adults over 60 because this is the population most likely to use MAID. In states where MAID is legal, 14.4% of respondents knew that MAID was legal in their state. In the full sample, 30% knew that MAID was legal anywhere in the United States. In the full sample, 20% reported that MAID was not morally acceptable, and 25% reported that they would probably or definitely not pursue MAID personally if they were faced with a terminal illness. This study revealed a significant gap in public awareness, with many residents unawares that they live in states where MAID is legal. However, attitudes towards MAID are mostly favorable with a 44% expressing support for MAID personally. These findings suggest the increased need for public education on MAID laws to ensure that residents are informed about their end-of-life-options.

